# Radiation Dose Reduction Opportunities in Vascular Imaging

**DOI:** 10.3390/tomography8050219

**Published:** 2022-10-21

**Authors:** David Summerlin, Joseph Willis, Robert Boggs, Loretta M. Johnson, Kristin K. Porter

**Affiliations:** 1 Department of Radiology, University of Alabama at Birmingham, Birmingham, AL 35249, USA; 2 School of Medicine, University of Alabama at Birmingham, Birmingham, AL 35249, USA; 3 Division of Physics and Engineering, University of Alabama at Birmingham, Birmingham, AL 35249, USA

**Keywords:** computed tomography angiography, magnetic resonance angiography, radiation dose, radiation reduction

## Abstract

Computed tomography angiography (CTA) has been the gold standard imaging modality for vascular imaging due to a variety of factors, including the widespread availability of computed tomography (CT) scanners, the ease and speed of image acquisition, and the high sensitivity of CTA for vascular pathology. However, the radiation dose experienced by the patient during imaging has long been a concern of this image acquisition method. Advancements in CT image acquisition techniques in combination with advancements in non-ionizing radiation imaging techniques including magnetic resonance angiography (MRA) and contrast-enhanced ultrasound (CEUS) present growing opportunities to reduce total radiation dose to patients. This review provides an overview of advancements in imaging technology and acquisition techniques that are helping to minimize radiation dose associated with vascular imaging.

## 1. Introduction

Patient radiation exposure is a known consequence of several common forms of medical imaging but has been deemed acceptable in a variety of conditions where the benefit of diagnosing or excluding an underlying medical condition outweighs the potential adverse effects of patient radiation exposure. Computed tomography (CT) and CT angiography (CTA) are the medical imaging methods with the highest radiation exposure, accounting for approximately half of the total radiation exposure in the United States (63% of which is attributed to CT scanning alone, according to the National Council on Radiation Protection and Measurements (NCRP) Report No. 184) [1]. While these imaging modalities deliver undesirable radiation exposure, the speed of acquisition, low cost, high sensitivity to a variety of pathology, and widespread availability/accessibility make CT (and CTA) the preferred imaging modality in a wide variety of clinical scenarios.

Current generations of CT scanners generate peak energies between 80 to 140 kV, with the majority using a tube potential of 120 kV, according to the International Commission on Radiological Protection (ICRP) Publication 135 [2]. The current one-year occupational radiation exposure limit of 50 millisieverts (mSv) was endorsed by the International Commission on Radiation Protection in 2007; however, the United States has not formally adopted these recommendations as of this writing [3]. Before the establishment of this threshold, both the Biologic Effects of Ionizing Radiation (BEIR) VII report and the American Association of Physicists in Medicine (AAPM) regarded 50 mSv as the maximum threshold for radiation exposure for a single procedure or 100 mSv for multiple procedures over a short period of time. It was felt that speculation of the harm caused to the patient by radiation may itself pose a more significant threat since, rather than receiving adequate medical care, patients may refuse potentially life-saving imaging [4,5].

A single CT examination can range anywhere from 1–30 mSv in adult patients depending on the type of scan, CT scanner, and the region of the body measured, while CTAs can reach 100 mSv in some cases [6]. While a single, standard CT is far below the proposed one-year cumulative limit of 50 mSv, it commonly exceeds the average annual latent exposure due to environmental radiation that one may experience in daily life, approximately 3 mSv annually [7]. Organizations such as the U.S (United States). Food and Drug Administration, United Nations Scientific Committee on the Effect of Atomic Radiation, and the International Committee on Radiological Protection all support the linear no-threshold (LNT) model, arguing that any amount of radiation may cause an increased rate of cancer [1]. The American College of Radiology (ACR) currently supports the ‘as low as reasonably achievable’ (ALARA) approach where no threshold recommendations are made, instead encouraging limiting radiation exposure when possible while also acknowledging that patients may require varying levels of radiation exposure to treat or diagnose the underlying condition appropriately. Multiple cross-disciplinary campaigns (including the Image Gently and Image Wisely campaigns for the pediatric and adult population, respectively) have been developed by the ACR, promoting the judicious use of imaging, and raising awareness regarding radiation exposure to the patient [8].

Commonly accepted radiation exposure limits for various examinations are developed by the ACR and must be validated regularly for accreditation for each CT scanner used at a given facility, termed the Computed Tomography Dose Index Volume (CTDI_vol_). Some example reference values of CTDI_vol_ include 25 milligray (mGy) for the adult abdomen, 15 mGy for the pediatric abdomen, and 75 mGy for the adult head [9]. Dose Length Product (DLP) is another method of characterizing exposure, representing a product of the CTDI_vol_ and the scanning length in centimeters (cm). Facility registries, such as the CT Dose Index Registry, as well as the development of guidelines (including the ACR Appropriateness Criteria), have been implemented to promote further radiation reduction throughout the medical imaging field [8]. Even with the various efforts and technological advancements made in recent years to reduce radiation exposure in medical imaging, patient cumulative radiation exposure still commonly exceeds the commonly referenced 50 mSv European recommended exposure limit.

Patients who undergo surveillance or postoperative scans are exposed to additional radiation even after the initial identification and intervention of the underlying medical condition. While ongoing surveillance scans are often indicated, the lifetime cumulative radiation exposure of continued surveillance scans may breach even the highest lifetime radiation exposure recommendations. Intraoperative exposure to radiation by fluoroscopy is another source of considerable radiation exposure used in a wide variety of procedures, including the guidance of intravascular catheters and for confirmation of orthopedic hardware placement. Organs with the highest tissue weighting factor (W_T_), a relative measure of the radiosensitivity of organs to radiation, include the stomach, colon, lung, bone marrow, and breast [10].

Some common surveillance scans that cause high levels of radiation exposure include annual cranial CTAs for post-stroke patients or aneurysm monitoring. Additionally, coronary CTA (CCTA) is commonly used to evaluate coronary artery disease (CAD) and is preferred over invasive coronary angiography for patients with low–intermediate risk of CAD [11]. The use of CCTA previously resulted in substantial radiation exposure of up to 18–31.4 mSv to achieve an adequate signal-to-noise ratio but has since been drastically reduced with the introduction of novel acquisition strategies where sub-millisievert (sub-mSv) acquisitions have been realized [12]. In patients with a history of substantial radiation exposure to the thorax, surveillance strategies may require additional consideration regarding the appropriate imaging modality in patients with heart disease [13].

As radiation reduction techniques continue to evolve in other areas of CT imaging [3,14,15,16], CTA remains a principal modality by which vascular pathology is identified and surveilled, prompting demand and interest in new radiation reduction strategies related to vascular imaging, including alternatives to the ionizing radiation associated with CTA. Magnetic resonance angiography (MRA) is one such alternative that has grown in clinical utility as technological advances have resulted in faster scan times, greater patient accessibility, potential for dynamic evaluation, and lower costs when compared to earlier iterations of MRA technology. Other alternatives have emerged in recent years, including contrast-enhanced ultrasound (CEUS), which offers benefits related to dynamic evaluation, lower cost, and, for some patients, increased tolerance (for patients who may struggle with claustrophobia, for example). Further advancements in CT, such as dual-energy CT (DECT), offer additional benefits over traditional acquisition and will likely become more widespread in the coming years. With the advent of these new imaging techniques, as well as increased awareness of radiation exposure overall, progress has been made as the annual individual effective dose of radiation from diagnostic and medical procedures has decreased by approximately 20% from 2.9 mSv in 2006 to 2.3 mSv in 2016 [17]. Artificial intelligence (AI) advancements demonstrate additional promise in lowering the radiation dose through computational techniques that improve image quality and reduce radiation exposure. The purpose of this review article is to evaluate current methods of radiation reduction in vascular imaging, including alternative imaging modalities such as MRA and CEUS, from a radiologist and physicist perspective when compared to CT or CTA.

## 2. Methods

A literature search was performed using the PubMed database and Google (Alphabet, Mountain View, CA, USA) search engine. Databases were searched using the keywords “radiation reduction techniques”, “magnetic resonance angiography radiation reduction”, and “computed tomography angiography radiation reduction”. Some specific searches for relevant topics such as the new MAGNETOM FreeMax (Siemens Healthineers, Erlangen, Germany) magnetic resonance imaging (MRI) system was performed using Google, as well as further exploration of topics around the use of AI in radiation reduction techniques.

## 3. Results

### 3.1. Computed Tomography Radiation Reduction Methodologies

The most efficacious strategies for radiation reduction adhere to the as low as reasonably achievable (ALARA) principle in which acquisition techniques are optimized to answer the clinical question, while reducing patient radiation exposure as much as possible [1].

#### 3.1.1. Image Acquisition Techniques

A simple but often overlooked method of radiation reduction includes reducing and/or optimizing the scan length, which has proven especially effective for radiation reduction in coronary CTA (CCTA) scans [1,18]. Scanning only the regions of interest can further reduce exposure by only imaging the clinically relevant areas of concern. Further radiation dose reduction is achieved by lowering the tube current modulation and voltage but at the detriment of the image quality due to increased noise [19,20].

Automatic tube current modulation (ATCM) enables CT scanners to alter tube current based on density differences in tissue attenuation, utilizing lower tube current when possible and subsequently resulting in lower radiation exposure [1]. Larger detector scanners (up to widths of 16 cm), as well as high pitch modes, are made possible via dual-source CT systems that can achieve sub-mSv exposure levels with the entire scan taking place during a single diastolic phase, further reducing radiation exposure [18].

#### 3.1.2. Image Reconstruction Techniques

New, sophisticated CT image reconstruction techniques require less patient radiation exposure to produce diagnostic level imaging quality. Advances in imaging reconstruction due to increased computer processing speeds allow for increased image processing complexity while retaining acceptable processing times.

Iterative reconstruction (IR) is one such example that reduces radiation exposure from 40–63% when compared to using filtered back projection (FBP) techniques for CCTA; new hybrid IR techniques are expected to further reduce dose exposure in the future [2,21,22,23]. Newer adaptive statistical iterative reconstruction (ASIR) takes advantage of additional parameters to reduce computational requirements compared to earlier IR techniques [3], further decreasing radiation exposure by up to 58%. IR algorithms vary by manufacturer and provide radiation dose reduction to various degrees but are frequently deployed in tandem with other radiation reduction techniques. IR is particularly useful for obese patients who require higher radiation exposure to achieve adequate imaging quality [24].

Other methods of radiation exposure reduction take advantage of the increased processing power now possible with modern computers, such as electrocardiogram (ECG)-controlled tube current modulation (ECTCM), a technique that reduces radiation exposure during periods of the cardiac cycle when the resulting image would be unused [25]. More sophisticated algorithms prospectively identify appropriate times for radiation dose reduction during CCTAs, further reducing the radiation exposure [18,22].

#### 3.1.3. Artificial Intelligence

AI shows promise as another method of radiation reduction through various methods but most notably in image reconstruction. Convolutional neural networks (CNN) are AI algorithms developed to recreate standard-dose images from low-dose computed tomography (LDCT) scans [26]. Deep learning image reconstruction (DLIR) uses neural networks to develop images with lower noise compared to FBP and ASIR images using 60% less radiation [27]. With a novel, modular approach that allows radiologists to view improvements in image quality iteratively, deep learning (DL) algorithms can improve the image quality of LDCT across multiple different vendors, with the resulting image quality rivaling normal dose computed tomography (NDCT) scans using traditional iterative reconstruction methods [28]. Other techniques, such as the Advanced Intelligent Clear-IQ Engine (AiCE), use DL during the process of reconstruction. These algorithms reduce the radiation dose of CTAs by up to 40% while also providing improved signal-to-noise and contrast-to-noise ratios [29]. Other CT vendors posit that DL reconstruction AI algorithms can reduce the radiation dose by up to 76% for abdominal CT images.

AI is also able to reduce radiation exposure by automating CTA tube voltage by dynamically selecting the appropriate voltage based on the patient’s anatomy and size, all while maintaining adequate image quality [2]. Another cause of excess radiation exposure involves the positioning of the patient within the CT gantry [29]. With new AI algorithms that utilize a depth camera to identify the proper position of a patient, some AI software is now used to properly position patients within the gantry and has been shown to reduce scan times as well as reduce radiation dose by up to 16%. Image reconstruction parameters for a variety of clinical indications are also being selected using AI algorithms. Even the selection of the correct exam may someday be performed with the assistance of AI, reducing unnecessary scans, ultimately resulting in reduced radiation exposure for the patient.

#### 3.1.4. Dual-Source CT

Radiation dose exposure reduction is also possible via the implementation of a dual-source CT (DSCT), which allows for faster image acquisition due to two detectors acquiring perpendicular images simultaneously while maintaining temporal resolution. DSCT is particularly effective in patients who cannot remain still [2]. Using DSCT in combination with IR allows for diagnostic quality imaging using less than 40 milliliters (mL) of contrast material, all while exposing the patient to less than 1 mSv. Furthermore, the third generation of DSCT can be utilized across most patients irrespective of their body habitus.

#### 3.1.5. Dual-Energy CT

Another advancement in the field of CT is dual-energy CT (DECT). DECT is particularly advantageous in the assessment of pulmonary embolus (PE) and the evaluation of chronic thromboembolic pulmonary hypertension (CTEPH) [30]. Studies show high sensitivity and specificity for the diagnosis of CTEPH at 100% and 92%, respectively, with DECT [31]. DECT involves acquiring a high and low-energy dataset that enables superior material decomposition and more accurate tissue characterization during image processing compared to single-energy CT. Perfused blood volume (PBV), a technique made possible by using DECT to create pulmonary iodine distribution maps, provides an objective assessment of perfusion defects, which enhances the diagnostic accuracy of PE detection. Furthermore, this technique may be a viable alternative to ventilation/profusion (V/Q) scans before lung transplantation [30].

Importantly, the photon counting detector in DECT machines can apply a threshold that enables the filtering of unwanted noise, allowing the scans to be performed at lower radiation doses [30]. Some studies have found that energy levels as low as 60 kiloelectronvolt (keV) may be optimal for thoracic imaging, down from peak energy ranging from 80 keV to 140 keV for standard CTs [2]. These methods also enable a reduction in contrast volume, which is advantageous for those with renal impairment and further reduces radiation exposure to the patient. Even under standard DECT imaging sequences, the radiation dose is no higher than a standard single-energy pulmonary CTA with a similar subjective image quality [2,32].

Virtual monoenergetic images (VMI), acquired using DECT, involve using either the projection domain or image domain to generate superior, blended images of high and low energy acquisitions, taking advantage of the high contrast offered in low keV scans while also benefitting from the low noise of the high keV acquisition [31]. VMI+, coined by applying the principle of VMI to dual source dual-energy acquisitions (the image domain variant of VMI), is made possible through DECT acquisitions. VMI (and VMI+) is particularly useful for vascular imaging, capitalizing on the benefits of high and low-energy scans allows for improved image quality in CT angiography by reducing the detrimental effects of iodine attenuation that often hampers diagnostic accuracy. VMI also improves the diagnostic accuracy of PE at lower energy levels (less than 60 keV), allows for superior reconstruction and monitoring of the thoracic aorta, and reduces the volume of contrast media volume needed to achieve diagnostic attenuation values. VMI has further utility when imaging around metal implants, reducing artifacts (such as beam hardening) with optimal photon energies of 105 to 120 keV.

DECT also offers an alternative to traditional contrast scans through virtual non-contrast images. These DECT scans are used to calculate the calcium score (CaSc) for coronary artery disease risk stratification. In the past, the level of radiation exposure via traditional angiographic imaging modalities led to this risk stratification method being downgraded [31]. Calcium subtraction images are another area where DECT is advantageous compared to traditional methods that require either time-consuming thresholding with manual corrections or double acquisition techniques, both of which led to increased radiation exposure compared to DECT. Dedicated DECT algorithms outperform thresholding and double acquisition methods by acquiring the images within a single CT phase. Virtual non-contrast images are also useful in evaluating aortic endovascular leak, with reductions in radiation exposure of greater than 60% via this method. Another potential benefit of DECT in vascular imaging is in identifying portal vein and deep vein thrombosis, where 40 keV virtual non-contrast images provide higher diagnostic confidence compared to linearly blended dual-energy CT scans according to a recent study [32].

DECT is also under investigation as a method of evaluating myocardial ischemia. Using color-coded-iodine perfusion maps, DECT scans can serve as an indirect perfusion marker and provide functional information regarding the patient’s coronary artery disease. Such information further increases the utility of DECT when compared to traditional CCTA, which only includes anatomic information [31]. Other studies suggest that DECT may be capable of visualizing myocardial fibrosis as well as late myocardial enhancement [32]. Further research is required to validate thresholds for establishing iodine uptake values, especially given the lack of standardization among DECT vendors.

### 3.2. Non-Ionizing Radiation Imaging Modalities

While many methods to reduce radiation exposure from CT or CTA scans have successfully limited radiation exposure, alternative imaging methods using non-ionizing imaging techniques can supplement, and in some cases replace, traditional irradiating scanning modalities. The ACR Appropriateness Criteria offers clinicians radiologist-approved imaging recommendations for various clinical indications, including a qualitative assessment of the amount of radiation exposure for a given technique, allowing clinicians to select viable alternative imaging methods which result in less or no radiation exposure [1].

#### 3.2.1. Magnetic Resonance Angiography

Magnetic Resonance Angiography (MRA) is a notable radiation-free alternative to CTA. Radiation exposure from CTA scans is significant, delivering some of the highest radiation dose exposure on a per scan basis of any medical imaging modality. MRA is capable of high-resolution cross-sectional, multiplanar imaging (often without contrast) in a wide variety of applications. In a meta-analysis comparing MRA with CTA, the results show that the two imaging modalities have the same diagnostic value for intracranial aneurysm evaluation, albeit with a limited sample size [33].

Both non-contrast MRAs (NC-MRAs) and contrast-enhanced MRAs (CE-MRAs) offer unique utility based on acquisition methods. Advantages of non-contrast studies include the absence of concern for nephrogenic systemic fibrosis (a condition associated with some of the gadolinium-based contrast agents that may be used in contrast-enhanced MRA), the relative simplicity and non-invasive nature of the scanning procedure compared to scans with contrast, and rapid repeatability of the scans which is particularly beneficial if initial scans are nondiagnostic due to motion artifacts or technical issues [34]. The ability to repeat scans also allows for acquiring images from multiple orientations if needed. Common limitations of non-contrast MRA include prolonged imaging times which result in an increased chance of motion artifacts and lack of information regarding collateral blood supply, which constrain the utility of this technique in the acute setting.

**Figure 1:**
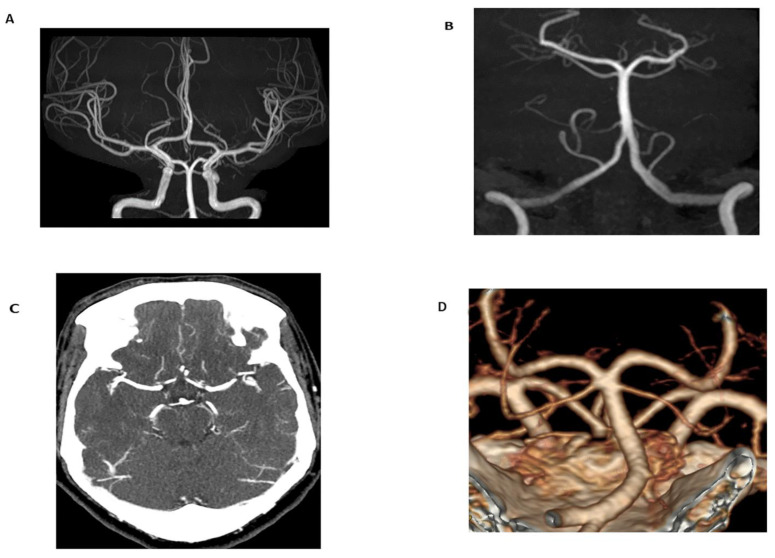
Time-of-flight (TOF) maximum intensity projection (MIP) image of the Circle of Willis (**A**) and basilar artery (**B**) in a 24-year-old female obtained for migraines and dizziness. The magnetic resonance angiogram (MRA) demonstrated no flow-limiting stenosis or occlusion. Additionally, imaging was performed without radiation or intravenous contrast (technical specifications: FOV 200.00 mm, TR 25 ms, TE 3.5 ms, 3 T magnetic field strength). In comparison to TOF-MRA, axial CT angiogram (CTA) MIP images (**C**) and volume-rendered images (**D**) in a 60-year-old female obtained for evaluation of tinnitus demonstrated no flow-limiting stenosis or occlusion. 100 mL of Omnipaque 350 contrast was administered and the CTDIvol for the CTA of the head and neck was 59.6 mGy.

Within the MRA imaging modality, multiple non-contrast techniques are available based on the region of interest. Flow-independent MRA is often utilized for imaging slow blood flow through veins and diseased arteries with a tradeoff in imaging quality from artifacts generated by off-resonant regions [34]. Flow-dependent MRA, on the other hand, consists of multiple different acquisition techniques, including time of flight MRA (TOF-MRA, Figure 1), inflow-dependent inversion recovery MRA (IFDIR-MRA), and quiescent-interval slice-selective (QISS) MRA. TOF-MRA is predominately a legacy MRA technique but is still widely used in imaging the extracranial arteries and the Circle of Willis. Recent advancements in TOF-MRA include compressed sensing TOF-MRA (CS TOF-MRA), which results in fewer motion artifacts by reducing scan times [35]. Using CS TOF-MRA for post-operative follow-up of patients with Moyamoya disease, CS TOF-MRA scans are superior to CTA based on a limited study, with a reduction in scan times of 50% (5 min 4 s compared to 10 min 8 s) when compared to traditional TOF-MRA methods. IFDIR-MRA, another flow-dependent MRA method, is primarily used for imaging of the renal arteries, providing diagnostic image quality in five minutes or less in patients with regular breathing patterns as the acquisition is typically acquired using respiratory-gated techniques [34].

For patients with renal dysfunction, QISS was developed to evaluate peripheral artery disease (PAD) without the need for contrast material. Requiring only 7 to 8 min on average for a whole-leg exam, QISS drastically reduces scan time when compared to the time of flight (TOF) methods that frequently take an hour or more for a whole-leg exam. QISS is also less prone to artifacts than TOF-MRA, and portions of the exam acquired with motion artifacts can easily be repeated when identified, resulting in only a 1-to-2-min prolongation of the exam time. QISS has also shown promise as a non-contrast, radiation-free imaging technique in diagnosing pulmonary embolism with a sensitivity and specificity of 86.0% and 93.3%, respectively, in a recent study [36]. The primary issues that occur with QISS revolve around a thicker minimum slice thickness resulting in reduced sharpness compared to other acquisition methods [34].

A third non-contrast MRA technique is subtractive 3D MRA. The cardiac-gated subtractive 3D fast spin-echo technique provides image quality comparable with CTA without the off-resonance artifacts that are prominent with flow-dependent MRA techniques. Other subtractive methods include flow-sensitive dephasing (FSD) and arterial spin labeling (ASL). FSD takes advantage of flow-dependent signal reductions between peak systole and end-diastole, resulting in arterial contrast after dephasing gradients are applied along the vessel length. ASL, another subtraction-based imaging acquisition method, is most commonly used as an MRA equivalent of x-ray digital subtraction angiography (DSA) for perfusion imaging of the brain but is also capable of imaging the extracranial carotid arteries. New developments in ASL imaging, such as 3D pointwise encoding time reduction MRA (PETRA-MRA), are in closer agreement with DSA when compared to TOF-MRA in the evaluation of intracranial stenosis [37]. Additionally, PETRA-MRA is notable for its superior image of intracranial stenosis both pre- and post-operatively when compared to TOF-MRA. Zero echo time radial ASL-MRA (zTE-MRA), which combines ASL methods with radial acquisition readouts, is another MRA technique that is superior to TOF-MRA in the evaluation of cerebrovascular disease [38]. zTE-MRA has the added benefit of noise-reduction during the acquisition of the exam, improving patient comfort compared to traditional TOF-MRA because of the lower scanning volume.

The final two notable techniques of non-contrast MRA acquisition include velocity-selective 3D MRA and phase contrast MRA. Velocity-selective MRA is beneficial in slow flow settings where alternative methods such as IFDIR and ASL-based MRA techniques may result in low penetration of tissues. A primary limitation of this method is that artifacts are prominent in a non-uniform magnetic field setting. Phase contrast is an advantageous technique in cardiac imaging, providing quantitative values for shunts and valvular disease. Phase contrast is unique amongst imaging modalities in that the more complex 4D (otherwise known as 3D cine) acquisitions are capable of simultaneously displaying vessel anatomy and flow rates. Like many other MRA techniques, phase contrast is primarily limited by lengthy scan times and the need for complex image processing.

**Figure 2:**
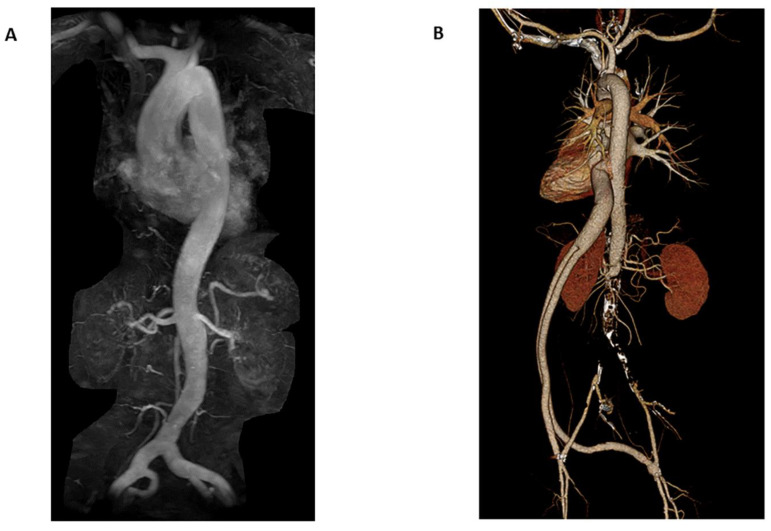
Coronal MIP MRA of the chest, abdomen, and pelvis (**A**) in a 66-year-old female for evaluation of possible aortic aneurysm demonstrates mild ectasia of the common iliac arteries bilaterally; however, no abdominal aortic aneurysm (AAA). 10 mL of Prohance contrast was administered. Coronal CTA volume rendered images (**B**) in a 70-year-old male with history of aortobifemoral bypass for suspected dissection demonstrates chronic occlusion of the infrarenal abdominal aorta and patent bypass. No dissection. 70 mL of Isovue contrast was administered with CTDIvol 19.2 mGy.

In some scenarios, contrast-enhanced MRA (CE-MRA) is a helpful technique for imaging flow-related phenomena (Figure 2). CE-MRA allows for better visualization of collaterals in acute ischemic stroke (AIS), which in turn is more predictive of the final infarct volume when compared to non-contrast TOF-MRA. Furthermore, the viability of collaterals correlates with improved outcomes in endovascular mechanical thrombectomy (EMT) [39]. Additionally, CE-MRA can characterize a variety of pathologies in which dynamic circulatory flow may be better seen with contrast. A specific CE-MRA technique, time-resolved (TR)-MRA, acquires and under samples k-space, allowing for the tracking of blood flow in a similar manner to conventional angiography. For example, in the evaluation and diagnosis of pelvic congestion syndrome, TR-MRA can generate studies that show contrast flowing into the renal vein before preferentially flowing retrogradely into the pelvis via the gonadal vein.

Cardiac MR (CMR) is an imaging modality commonly used for long-term surveillance of adult congenital heart disease (ACHD), which is preferred over alternative methods due to the lack of radiation as well as the ability to avoid the rib spaces (which can be challenging with transthoracic echo) [40]. Compared to other alternatives, CMR is better suited to assess valvular function, flow, and shunting [11]. When evaluating for significant coronary artery stenosis, CMR angiography (CMRA) is more specific than CTA. CMRA offers a radiation and contrast-free alternative for the evaluation of CAD, particularly for patients who can maintain a heart rate of less than 70 beats per minute. Additional benefits, including the simultaneous acquisition of anatomical and functional data combined with the lack of calcium blooming artifact, make CMRA a preferable choice in a variety of scenarios, including patients with chronic renal failure who are more prone to high coronary calcification burden [41].

Regarding MRA performance, a systemic meta-analysis demonstrates sensitivity and specificity of 91% and 88%, respectively, for TOF-MRA of the extracranial arteries [42]. However, in the same study, the sensitivity for detecting moderately severe 50–69% internal carotid artery stenosis is only 38% (specificity of 92%). A review by Edelman and Koktzoglou compares many studies that report sensitivities and specificities of MRA intracranial vascular stenosis ranging from 80–88% and 87–97%, respectively, which is on par with either DSA or CTA as controls [34]. A more recent review article found no difference in intracranial aneurysm detection between TOF-MRA and CTA, with MRA having a slightly lower sensitivity of 80% compared to 84% for CTA and high specificity at 87% compared to CTA at 85% [33]. Regarding cardiac imaging, MRA, using appropriate cardiac-gated protocols with whole-heart coverage, has a sensitivity of 87% and a specificity of 69%. For the detection of pulmonary embolic, sensitivity is 85%, compared to a specificity of 98% for CTA. In evaluating renal artery stenosis via IFDIR protocols with a 1.5 Tesla magnet, a sensitivity of 88% and a specificity of 95% are reported for CE-MRA, DSA, or CTA. Celiac trunk and mesenteric artery stenosis with IFDIR yields accuracies of 89% and 95%, respectively. Portal vein, hepatic artery, and hepatocellular carcinoma scans are promising with early results indicating that IFDIR may be a beneficial method of imaging these pathologies, but further study is required.

#### 3.2.2. Open and Larger Bore MRI

One common barrier to utilizing MRA as an alternative to CTA revolves around the availability and/or accessibility of scanners, as well as the relative difficulty that many claustrophobic patients experience due to the traditional scanner design. The prolonged scanning times of MRAs compared to CTAs create additional difficulty for some patient groups. With recent advancements in MR design and reconstruction, new scanners are attempting to mitigate many of the issues associated with MR, including increasing the bore size to accommodate larger patients and reduce claustrophobia. For example, the FDA recently approved a 0.55 Tesla (T) magnet with a larger bore design (80 cm). Importantly, this product has substantial cost savings compared to other scanners due to the lower field power, which allows the scanner to be lighter and easier to transport [43]. Additionally, the scanner does not require quench pipes or helium refills, reducing facility and operational costs. The large-bore design allows claustrophobic patients to undergo scans while also benefiting the pediatric population, where children can be accompanied by a caregiver while undergoing the scan. Another beneficial aspect of the low-field scanner is that the noise level of the scanner is reduced compared to a standard MRI due to the lower field strength, leading to a quieter and more comfortable scan for the patient.

While poor resolution has traditionally been associated with lower-powered magnets, some aspects of a low-field strength are beneficial to the image acquisition process [43]. Low-field strength magnets benefit from a more uniform, larger magnetic field. With a shorter T1 relaxation time due to the lower strength field, the recently FDA approved 0.55 T magnet is roughly 25% faster than a comparable 1.5 T magnet [44]. As a result, multiple images can be averaged to achieve image signal-to-noise ratio (SNR) parity at 2.25 times the imaging time compared to triple the imaging time. A reduction in susceptibility artifacts and less geometric distortion can also lead to improved imaging of metallic implants and the optic nerves, particularly for diffusion-weighted imaging (DWI) [43,44]. With a lower Larmor frequency because of the lower strength magnetic field, the associated RF wavelength is longer, resulting in reduced interference and overall improved homogeneity of the field [44].

#### 3.2.3. Contrast-Enhanced Ultrasound

**Figure 3:**
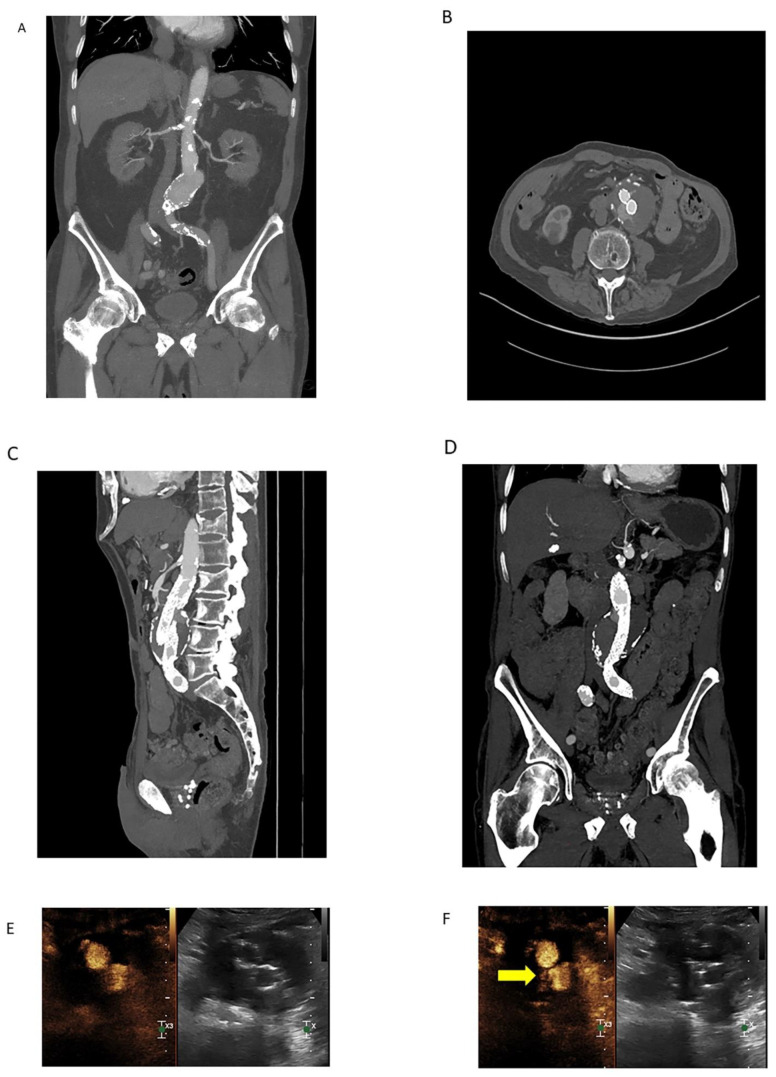

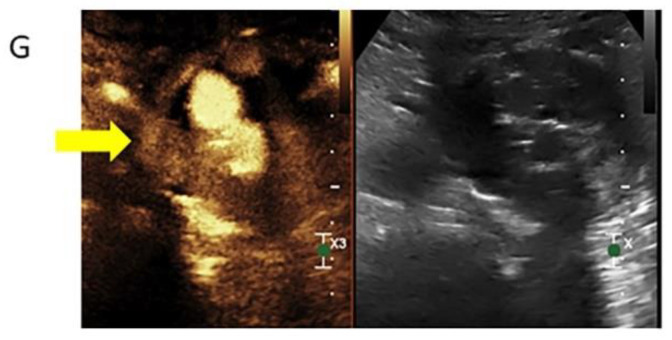
Coronal MIP image (**A**) demonstrating an infrarenal AAA in a 78-year-old male obtained prior to endovascular repair. Calcific atherosclerotic plaque and vessel patency well-demonstrated on this arterial phase CTA. 100 mL of Omnipaque 350 contrast was administered with CTDIvol of 7.68 mGy. Arterial phase source axial image (**B**) from a CTA demonstrating endovascular repair of the infrarenal AAA with persistent contrast flow into the excluded aneurysm sac, consistent with an endoleak. Sagittal (**C**) and coronal (**D**) MIP images are also shown. Lack of dynamic imaging limits classification of the type of endoleak; although, it was initially characterized as a type II endoleak by CTA. 100 mL of Isovue 370 contrast was administered with CTDIvol of 5.77 mGy. Subsequent contrast-enhanced ultrasound (CEUS, (**E**)) with dynamic contrast enhancement demonstrated a type III endoleak arising from the origin of the left iliac limb ((**F**), yellow arrow). The type III endoleak represents the major component and a large portion of the bolus empties into the aneurysm sac ((**G**), yellow arrow) with a small type II contribution arising from a right lumbar artery (not shown). Three boluses totaling 5 mL of Lumason were injected.

Contrast-enhanced ultrasound (CEUS) has many advantages over other imaging modalities, including low-cost, non-nephrotoxic contrast agents and a low rate of complications compared to iodinated contrast agents [45]. CEUS is effective in identifying endothelial surface disruptions such as plaque ulcerations and may be a viable method of monitoring carotid plaques, especially when combined with duplex ultrasound (DUS) [45]. CEUS improves the diagnostic accuracy of critical limb revascularization compared to DUS alone, and CEUS is more effective in EVAR surveillance compared to CTA or DUS in isolation, particularly with detecting specific types of leaks (Figure 3). Due to the lack of radiation exposure, continuous scanning of the region of interest is possible, negating the theoretical risk of missing a delayed extravasation [46].

Dynamic feedback, a feature unique to the ultrasound (and CEUS) modality, allows for real-time feedback that is particularly advantageous in targeted biopsies where examiners can distinguish between vascularized and non-vascularized tissue [47]. This type of feedback is useful when placing drains, enabling drain position verification and assessment at bedside, with rapid position confirmation when repositioning of the drain is required. The ability to attain multiplanar views from various orientations allows for repositioning as indicated to optimize the approach while avoiding adjacent vasculature near the intervention site [46]. CEUS is also useful in ablative therapies where small lesions are more visible with the addition of contrast [47]. In the postoperative setting, CEUS can be used to monitor lesions as the exam is rapidly repeatable and therefore provides an imaging modality capable of confirmatory imaging over time to complete resolution [48]. High- and low-energy trauma can benefit from CEUS, especially in the pediatric population, to prevent unnecessary radiation exposure while enabling the use of follow-up exams over time.

Important limitations of CEUS include the intralesional gas formation, which limits the utility of ultrasound. Ultrasound utility is limited in areas where the acoustic window may be obstructed by rib shadows which may be further complicated by an intervening bowel gas as well as patient respiration and immobility [46]. Furthermore, the ability of CEUS intervention is determined to a large degree by operator skill level and experience, especially when compared to alternative imaging modalities such as CT and MRI.

### 3.3. Other Methods of Radiation Dose Reduction

As discussed previously, a variety of techniques have been developed to reduce radiation exposure that focus on the scanning modality itself. While these methods have been well-studied, other methods of radiation reduction that are unrelated to the imaging modality are effective and may be more easily to implement, especially when financial limitations are a limiting factor when attempting to reduce radiation exposure.

#### 3.3.1. Alternating Imaging Modalities

In cases where regular surveillance of vascular pathology is required, performing surveillance scans using an approach that alternates between irradiating (CTA) and non-irradiating (MRA) imaging modalities may be a reasonable method to reduce radiation exposure in high-risk patients. As previously described in a publication regarding endovascular repair surveillance [49], alternating imaging modalities offers a favorable combination of reduced radiation exposure, decreased cost, and increased availability over using either CTA or MRA in isolation. The use of alternating modalities may be an underutilized method of radiation reduction as a dearth of information regarding such imaging protocols currently exists.

#### 3.3.2. Protocol Driven Radiation Dose Reduction

The creation and implementation of CT imaging protocols requires a team approach, often made up of radiologists, physicists, and CT technologists. Studies have shown that the more individuals involved in the process, the more variable the radiation dose. Employing external medical physicists is associated with increased radiation exposure in lung cancer screening (LCS) exams compared to internal medical physicists, while using any type of medical physicist is associated with lower radiation exposure overall [15]. Furthermore, institutions in which a designated radiologist approves protocols are associated with a lower level of radiation exposure compared to allowing any radiologist to approve protocols. Selection of the appropriate imaging protocol is another component of protocol drive radiation dose reduction in which clinician decision support software provides opportunities to assist clinicians in the process of selecting the optimal imaging study and protocol. For example, a new aortic dissection protocol for patients in the emergency department (ED) can reduce radiation exposure by up to 14.6% according to a recent study [50]. This same protocol is also responsible for a 16% reduction in contrast volume in the same population.

### 3.4. High-Risk Populations

Patients at risk of high lifetime radiation exposure should receive special consideration regarding imaging modality selection, as many may require long-term, regular screening for potentially life-threatening pathology.

#### 3.4.1. Children

In children, radiation reduction is of paramount importance due to the detrimental effects that cumulative radiation exposure may have on a child’s development and risk of cancer. Kawasaki disease, which requires monitoring for evaluation of coronary aneurysms, can result in high radiation exposure in children as coronary CTA has been the predominant surveillance method [41]. The Japanese Circulation Society guidelines support using coronary MRA over coronary CTA for monitoring Kawasaki patients to reduce radiation exposure, a recommendation initially made by the society in 2013. Children born with congenital heart malformations constitute another population that may require post-operative imaging or regular surveillance, making imaging non-irradiating modalities desirable. Additional conditions in which children may require repeated vascular imaging include the evaluation of arterio-venous malformations and children with Turner syndrome for monitoring of coarctation of the aorta and bicuspid aortic valve.

#### 3.4.2. Marfan Syndrome

Genetic aortic syndromes, the most common of which is Marfan syndrome, are genetic conditions that require regular imaging for monitoring of the aortic diameter, which is an important predictor of potentially life-threatening aortic aneurysms and aortic dissections [51]. As a result, these patients could potentially be exposed to a high cumulative radiation dose over their lifetime depending on the frequency of imaging as well as the chosen imaging modality. CTA is the reference standard for ruling out aortic dissection (particularly in the acute setting) and provides high-resolution imaging for accurate aortic measurements but is generally not recommended for routine surveillance due to the high radiation exposure. Transthoracic echocardiography (TTE) provides adequate aortic and valvular views to assess aortic function. Current recommendations for monitoring Marfan syndrome include a TTE at diagnosis with regular 6-month assessments of the aortic root and proximal ascending aorta, which can be lengthened to annual exams if the aorta is found to be stable in size. However, many patients with the associated genetic syndromes such as Marfan syndrome have challenging body habitus (such as Pectus excavatum in Marfan syndrome), which makes acquiring diagnostic views using TTE difficult. Non-contrast MRA is equally effective to CTA in evaluating aortic diameter and is non-invasive, forgoing any challenging anatomy compared to the transthoracic echocardiography approach, with the primary drawback of non-contrast MRA being increased exam time. Phase contrast MRI is a relatively new imaging modality closely related to MRA that has shown promise for monitoring aortic syndromes. Using this image acquisition method, dynamic blood flow profiles are generated to better predict complex hemodynamic changes in the vessels of these patients and provide a more complete assessment of the forces acting on the aortic wall [51]. With this information, early identification of pathological changes in blood flow may allow for earlier intervention in patients with aortic syndromes, but further research is needed to quantify the prognostic values of these techniques.

#### 3.4.3. Ehlers-Danlos Syndrome

Vascular Ehlers-Danlos (previously Ehlers-Danlos type IV) is another aortic syndrome that requires special consideration because of the potential for high radiation exposure. Although no evidence-based guidelines have been developed, institutions have a range of screening recommendations for patients with Vascular Ehlers-Danlos, ranging from regular screening for vascular abnormalities of the arterial tree to a single transthoracic echo in adults with no further follow-up [51,52]. Doppler ultrasound, CTA, or MRA are the predominant modes of screening in Vascular Ehlers-Danlos [52].

#### 3.4.4. Loeys-Deitz Syndrome

Loeys-Deitz syndrome, another aortic syndrome, overlaps with Marfan syndrome and/or Vascular Ehlers-Danlos, depending on the specific subtype [51]. Due to a higher propensity for abdominal or intracranial aneurysms than other aortic syndromes, screening recommendations for Loeys-Deitz syndrome include CTA/MRA of the entire aorta at diagnosis with fully body MRA recommended every one to two years depending on exam findings.

#### 3.4.5. Turner Syndrome

Turner syndrome is another condition in which cardiovascular imaging is recommended for bicuspid aortic valve and aortic isthmus stenosis, two congenital cardiovascular malformations that are commonly seen with Turner syndrome [51]. The recommended imaging frequency varies based on risk stratification from every 6 months in high-risk patients to every 10 years for patients with low concern for cardiovascular complications. TTE and MRA are the preferred imaging modalities for assessment.

#### 3.4.6. Bicuspid Aortic Valve

Bicuspid aortic valve, independent of patients with Turner syndrome, is the most common cardiovascular malformation with a prevalence of 1000–2000/100,000 [51]. Patients with bicuspid aortic valve require regular monitoring for aortic dissection, with TTE being the preferred imaging modality of choice according to the 2018 American Association for Thoracic Surgery Guidelines, while MRA or CTA is the imaging modality of choice when the proximal aorta diameter exceeds 4.5 cm [53].

#### 3.4.7. Other High-Risk Populations

Outside of genetic conditions, many patients may have more common conditions that require special consideration regarding lifetime radiation exposure. Radiation therapy can be a life-saving treatment for patients with a variety of malignancies, however the impact of cumulative dose should be considered when imaging these patients [18]. Other patients at risk for high radiation exposure are patients with recurrent abdominal pain who may undergo repeat CT scans out of precaution by providers [1]. In one study, 2% of patients who had a CT in the emergency department had three or more CTs within the past seven years with a cumulative radiation dose of 120 mSv. Patients with renal colic are another patient subset who regularly undergo repeat CTs. Coronary artery risk stratification using CTA may expose patients to high levels of radiation if the calcium burden is monitored continuously over time [31]. Other cardiac conditions with high radiation exposure potential are patients who are monitored for endovascular leaks after endovascular aortic aneurysm repair. Furthermore, patients with any metallic implants require higher radiation levels when the implant is within the field of view, leading to elevated radiation exposure relative to those without metallic implants, especially when these implants are in an area of concern for additional pathology.

## 4. Discussion

Through various advancements both in imaging acquisition and processing, the average radiation dose has drastically fallen over the last decade. Various institutions have successfully established protocols that reduce radiation through a combination of decision-making algorithms and careful coordination amongst multiple clinicians involved in the individual’s care. CT technique remains the modality of choice for most indications related to vascular imaging. However, several advancements both with CT technology as well as alternative imaging modalities have contributed to opportunities for significant reductions in radiation exposure (Table 1).

DECT allows for more accurate tissue characterization while at the same time requiring lower maximum energy ranges when compared to traditional CT scans. Through this novel technique, a wide range of clinical applications show promise when imaged with DECT, including lung and myocardial perfusion studies, calcium subtraction imaging for coronary artery disease evaluation, detection of portal and deep vein thrombosis, and PE detection, all with benefits of lower or equivalent radiation exposure without the need for exogenous contrast administration.

The development of the low-field MRI offers a radiation-free alternative that will gradually become more accessible to a larger subset of the population. By reducing setup costs and other barriers through the novel design of new scanners, MRI may become the imaging modality of choice in the coming years for a wide variety of clinical indications and various screening exams. In specific situations, such as the intensive care unit (ICU) and ED, smaller, portable MRI systems have been shown to be effective, improving efficiency by reducing personnel required due to the portability of the scanner while also improving patient care with a bedside solution that negates the potentially detrimental transportation of the patient that would have previously been a necessity.

MRA has proven to be a reliable alternative for radiation reduction, particularly in scenarios where CTA may be traditionally deployed. Multiple studies have found that MRA is comparable to CTA in diagnosing various vascular pathology while offering superior image quality in certain situations such as in the setting of severe atherosclerosis and some metallic artifacts. New techniques allow for the dynamic assessment of blood flow in evaluating pathologic changes in the vessels. Other methods reduce scan time, further reducing the time discrepancy that remains a predominant critique of MRA compared to CTA’s rapid scan times. While CTA will likely remain the predominant modality of choice, advancements in MRA acquisition techniques continue to narrow the gap between MRA and CTA, offering a capable and radiation-free alternative for a variety of indications.

Another promising radiation reduction development is deploying AI and scanner learning to acquire low-dose, diagnostic quality scans. With rapid advancements in DL algorithms, AI can reduce radiation exposure by improving the image quality of low-dose images while also reducing processing times compared to traditional iterative reconstruction techniques. Outside of the image quality domain, AI can improve patient positioning during the scan, optimizing the patient’s location within the scanner which reduces radiation exposure. The diagnostic yield of various exams can also be enhanced through AI, allowing clinicians to improve their decision-making process by leveraging the large volumes of data available.

While reducing radiation exposure through various hardware and software advancements will continue to reduce total exposure, improving the decision-making process of ordering scans and reducing unnecessary scans is another method of reducing radiation exposure that should be considered. Making decision-making support systems using information technology available for clinicians increases the diagnostic yield of the scans ordered while also reducing both the exposure to the patient and the volume of scans at the medical center [54].

## 5. Limitations

Unfortunately, many medical imaging facilities continue to utilize legacy scanners that limit access to modern radiation reduction techniques. DECT-capable scanners have not been widely adopted as many facilities continue to use legacy CT scanners. MRI accessibility is even more limited due to the higher cost associated with this technology. The development of new image acquisition methods that could be applied to existing scanners to improve the diagnostic quality of low-dose scans may prove useful in situations where new equipment may be cost-prohibitive or financially impractical.

Numerous benefits of CT often prevent consideration of other modalities. These benefits include the widespread availability of CT scanners in most hospitals (including smaller, rural locations) as well as other factors such as staffing and scan duration [43,55]. This discrepancy is particularly evident in the trauma or emergency setting, where CT technologists are available at all hours while many MRI and ultrasound staff have more limited availability. When catheter access is required for intervention, CTA, due to its rapid speed of acquisition, has the benefit of acquiring imaging of larger segments of the body during the initial evaluation, allowing for careful planning of any potential endovascular procedure, whereas using MRA or ultrasound for such planning would be less time efficient.

Given the widespread availability of CT and a variety of other factors already discussed, many trials and research studies have focused on CTA’s diagnostic performance and efficiency rather than MRA or other techniques. For example, CTA was the predominant imaging modality used to identify proximal large-artery occlusion in various trials where mechanical thrombectomy was shown to be superior to noninterventional approaches [55]. CTA also remains the gold standard in evaluating intracranial hemorrhage (ICH) due to its higher sensitivity, positive predictive value, and inter-rater reliability compared to MRA [55]. However, given rapid advances in a variety of MR techniques, further studies are needed to examine and/or re-examine the sensitivity and specificity performance of MRA compared to CTA for various clinical indications.

The development of AI for widespread use also raises some concerns. Lack of verifiability across imaging platforms, as well as the subjective human ratings that are relied upon during the approval process for AI deployment, have been called into question [29]. Collaboration amongst all parties involved, including clinician investigators, vendors, and software developers, is needed to ensure that AI development is applicable across a wide range of use-cases [26].

## 6. Conclusions

Vascular imaging constitutes a robust volume of imaging in many modern radiology practices, ranging from smaller private practice settings to larger referral centers. Traditional methods for evaluating vascular pathology often involved high levels of radiation exposure to patients; however, modern techniques and advances in vascular imaging have significantly reduced radiation doses associated with this segment of diagnostic imaging. A variety of dose reduction techniques and strategies have been developed and applied to CTA imaging which remains the most accessible method to evaluate vascular pathology. Recent advances in alternative non-ionizing imaging modalities have either reduced and/or eliminated differences in sensitivity and specificity between CTA and alternative modalities. However, cost and accessibility remain potential obstacles to realizing the widespread use of these alternative modalities. In particular, technological advances in the sector of MRA imaging have been so rapid that research evaluating performance compared to CTA has been slow to catch up. As researchers further evaluate and confirm the performance of these advances in vascular imaging for both ionizing and non-ionizing radiation imaging techniques, radiologists and clinicians should expect patient radiation exposure associated with imaging of vascular pathology to continue to decrease.

## Figures and Tables

**Table 1:** Radiation Dose Reduction Techniques.

Computed Tomography Radiation Reduction Methodologies	
Image Acquisition Techniques	- optimization of scan length, voltage - tube current modulation
Image Reconstruction Techniques	- iterative reconstruction - electrocardiogram-controlled tube current modulation
Artificial Intelligence	- deep learning reconstructive techniques - dynamic tube voltage modulation - patient positioning
Dual-source CT	- faster acquisition
Dual-energy CT	- superior tissue characterization - thresholding for noise reduction - reduced contrast volume required - virtual monoenergetic images - perfusion mapping
**Non-Ionizing Radiation Imaging Modalities**	
Magnetic Resonance Angiography	- alternative to CTA - high-resolution - iodinated contrast sparing - capable of functional/dynamic imaging
Cardiac MR/MRA	- avoids rib spaces - resistant to image degradation of high coronary calcium burden - iodinated contrast sparing - functional/dynamic imaging
Low-field MRI	- decreased cost (relative to standard MRI) - decreased susceptibility - more suitable for claustrophobic/pediatric population
Contrast-Enhanced Ultrasound	- low cost - dynamic imaging - real-time feedback - unlimited imaging orientations
**Other Methods of Radiation Reduction**	
Alternating Imaging Modalities	- reduces radiation exposure by ½ - cost advantageous over MRA alone - increased availability over MRA alone
Protocol Driven Radiation Dose Reduction	- hiring of medical physicists proven to be associated with lower patient exposure - systemwide radiation reduction via protocols
Low Frame Rate Fluoroscopy	- radiation reduction method in high-exposure modality - may be compatible with existing equipment

## Data Availability

The data presented in this study are available on request from the corresponding author.

## Abbreviations

AAA	abdominal aortic aneurysm
AAPM	American Association of Physicists in Medicine
ACHD	adult congenital heart disease
ACR	American College of Radiology
ACTM	automatic tube current modulation
AI	artificial intelligence
AIS	acute ischemic stroke
AiCE	Advanced intelligent Clear-IQ Engine
ALARA	as low as reasonably achievable
ASIR	adaptive statistical iterative reconstruction
ASL	arterial spin labeling
BEIR	Biologic Effects of Ionizing Radiation
CAD	coronary artery disease
CaSc	calcium score
CCTA	coronary computed tomography angiography
CEUS	contrast-enhanced ultrasound
CE-MRA	contrast-enhanced magnetic resonance angiography
cm	centimeter
CMR	cardiac magnetic resonance
CMRA	cardiac magnetic resonance angiography
CNN	convolutional neural networks
CS TOF-MRA	compressed sensing time of flight magnetic resonance angiography
CT	computed tomography
CTA	computed tomography angiography
CTDI_vol_	Computed Tomography Dose Index Volume
CTEPH	chronic thromboembolic pulmonary hypertension
DECT	dual-energy computed tomography
DL	deep learning
DLIR	deep learning image reconstruction
DLP	dose length product
DSCT	dual-source computed tomography
DWI	diffusion weighted imaging
DUS	duplex ultrasound
ECG	electrocardiogram
ECTCM	electrocardiogram-controlled tube current modulation
ED	emergency department
EMT	endovascular mechanical thrombectomy
FBP	filtered back projection
FPS	frames per second
FSD	flow-sensitive dephasing
ICH	intracranial hemorrhage
ICRP	International Commission on Radiological Protection
ICU	intensive care unit
IFDIR-MRA	inflow-dependent inversion recovery magnetic resonance angiography
IR	iterative reconstruction
keV	kiloelectronvolt
LCS	lung cancer screening
LDCT	low dose computed tomography
mGy	milligray
mL	milliliter
MRI	magnetic resonance imaging
MRA	magnetic resonance angiography
mSv	millisievert
NC-MRA	non-contrast magnetic resonance angiography
NCRP	National Council on Radiation Protection and Measurements
NDCT	normal dose computed tomography
PCI	percutaneous coronary intervention
PE	pulmonary embolus
PETRA-MRA	pointwise encoding time reduction magnetic resonance angiography
QISS	quiescent-interval slice-selective
SNR	signal to noise ratio
T	Tesla
TOF	time of flight
TOF-MRA	time of flight magnetic resonance angiography
TTE	transthoracic echocardiography
V/Q	ventilation/profusion
VLFF	very low frame rate fluoroscopy
VMI	virtual monoenergetic images
W_T_	tissue weighting factor
zTE-MRA	zero echo time magnetic resonance angiography
